# L-Citrulline Supplementation Increases Plasma Nitric Oxide Levels and Reduces Arginase Activity in Patients With Type 2 Diabetes

**DOI:** 10.3389/fphar.2020.584669

**Published:** 2020-12-22

**Authors:** Alia Shatanawi, Munther S. Momani, Ruaa Al-Aqtash, Mohammad H Hamdan, Munir N. Gharaibeh

**Affiliations:** ^1^ Department of Pharmacology, School of Medicine, The University of Jordan, Amman, Jordan; ^2^ Department of Internal Medicine, School of Medicine, The University of Jordan, Amman, Jordan; ^3^ Department of Neurosurgery, Saarland University Hospital, Homburg, Germany

**Keywords:** arginase, nitric oxide, citrulline, arginine, diabetes, vascular function

## Abstract

Type 2 diabetes mellitus (T2DM) is becoming a major contributor to cardiovascular disease. One of the early signs of T2DM associated cardiovascular events is the development of vascular dysfunction. This dysfunction has been implicated in increasing the morbidity and mortality of T2DM patients. One of the important characteristics of vascular dysfunction is the impaired ability of endothelial cells to produce nitric oxide (NO). Additionally, decreases in the availability of NO is also a major contributor of this pathology. NO is produced by the activity of endothelial NO synthase (eNOS) on its substrate, L-arginine. Reduced availability of L-arginine to eNOS has been implicated in vascular dysfunction in diabetes. Arginase, which metabolizes L-arginine to urea and ornithine, competes directly with NOS for L-arginine. Hence, increases in arginase activity can decrease arginine levels, reducing its availability to eNOS and decreasing NO production. Diabetes has been linked to elevated arginase and associated vascular endothelial dysfunction. We aimed to determine levels of plasma NO and arginase activity in (T2DM) patients and the effects of L-citrulline supplementation, a natural arginase inhibitor, on inhibiting arginase activity in these patients. Levels of arginase correlated with HbA1c levels in diabetic patients. Twenty-five patients received L-citrulline supplements (2000 mg/day) for 1 month. Arginase activity decreased by 21% in T2DM patients after taking L-citrulline supplements. Additionally, plasma NO levels increased by 38%. There was a modest improvement on H1Ac levels in these patients, though not statistically significant. The effect of L-citrulline on arginase activity was also studied in bovine aortic endothelial cells (BAECs) grown in high glucose (HG) conditions. HG (25 mM, 72 h) caused a 2-fold increase in arginase activity in BAECs and decreased NO production by 30%. L-citrulline (2.5 mM) completely prevented the increase in arginase activity and restored NO production levels. These data indicate that L-citrulline can have therapeutic benefits in diabetic patients through increasing NO levels and thus maintaining vascular function possibly through an arginase inhibition related pathway.

## Introduction

Type 2 diabetes mellitus (T2DM) is becoming one of the leading global health problems. By 2030 more than 400 million people are expected to have diabetes. Many of these patients will suffer from the complications of diabetes (Bommer et al., 2018). Uncontrolled diabetes can lead to serious complications if not properly managed. Mostly, these are either microvascular complications including nephropathy, neuropathy and retinopathy. or macrovascular pathologies such as coronary artery disease, peripheral artery disease and cerebrovascular disease (Deedwania, 2004).

Additionally, insulin resistance associated with T2DM can lead to inflammation and vascular dysfunction leading to accelerated atherosclerosis, and to elevated oxidative stress in the body. One of the important characteristics of vascular dysfunction in diabetes is reduction in nitric oxide (NO) availability (Romero et al., 2012).

NO is considered a key regulator of vasomotor tone and function under physiological and pathological conditions (Alderton et al., 2001). It has been recently shown that NO has an important role in modulating insulin sensitivity and glucose disposal (Xia et al., 1996; Caldwell et al., 2010). Additionally, in conditions of hyperglycemia and insulin resistance, NO synthase (NOS) activity has been shown to be impaired. (Rask-Madsen & King, 2007). Low levels of arginine can lead to NOS uncoupling, which can enhance reactive oxygen species production instead of NO leading to oxidative stress (Xia et al., 1996).

Recent studies have shown a clear involvement of arginase in diabetic cardiovascular, retinal, renal and neuronal complications (Caldwell et al., 2010). Arginase, a hydrolytic enzyme that metabolizes the semi-essential amino acid, L-arginine, is distributed in tissues throughout the body in two isoforms and has functions in both health and disease. In the liver, this enzyme is a key element of the urea cycle to dispose f harmful urea. This cycle removes toxic ammonia, formed through protein catabolism (Morris Jr, 2009). In the vascular system, when activated, arginase can compete with nitric oxide synthase (NOS) for their common substrate, L-arginine. Decreases in L-arginine availability to NOS can lead to decreased production of NO, and possible NOS uncoupling and increased superoxide formation (Berkowitz et al., 2003).

L-citrulline is an amino acid available in watermelon, specially its rind (Mandel et al., 2005). It exerts beneficial effects on the cardiovascular system by supporting enhanced NO production (Osowska et al., 2004). In isolated blood vessel preparations, L-citrulline induced endothelial dependent relaxation by enhancing the release of NO. This NO release could be due to the recycling of L-citrulline to L-arginine (Raghavan and Dikshit, 2001). Moreover, L-citrulline also seems to have beneficial effects on retinal arteries, also mediated through NO pathway (Mori et al., 2015).

While its essential to remember that in the vascular endothelium, NO is produced by endothelial NO synthase (eNOS), which uses L-arginine as a substrate and produces L-citrulline as a by-product, it is also important to mention that the L-citrulline to L-arginine recycling pathway involves two enzymes, argininosuccinate synthase and argininosuccinate lyase, which are present in endothelial cells and other cell types. Interestingly, L-citrulline has also been shown to possess an inhibitory effect on arginase (Shearer et al., 1997). This inhibitory effect can further augment the production of NO through providing more L-arginine for the NOS pathway (Romero et al., 2006).

The purpose of this study is to examine the effects of L-citrulline supplementation on arginase activity and NO levels in type-2 diabetic patients. Additionally, the effects of L-citrulline on endothelial cells and on blood vessels were studied in bovine aortic endothelial cells and in mice aortas *ex-vivo.*

### Subjects and Methods

#### Patient Selection and Testing

This study is an interventional non placebo control trial registered at ClinicalTrials.gov with the Identifier: NCT03358264. Study included 25 type 2 diabetic patients attending the Endocrine Clinic at Jordan University Hospital (JUH). Patients, 25–65 years-age, with type 2 diabetes on oral hypoglycemic drugs were selected. Each patient signed an informed consent form to participate in the study that was approved by the JUH Ethics Committee. Procedures were followed in accordance with the Helsinki Declaration of 1975. Patients taking insulin were excluded from the study. Patients were interviewed for a full medical history and a list of concomitant medications.

Blood samples (3–5 ml) were collected in ethylenediaminetetraacetic acid (EDTA) tubes for arginase activity, glycated hemoglobin (HbA1c), fasting blood sugar and nitrites measurement. All samples were kept at −4 °C and processed in the same day.

### Plasma Sample Preparation

Cooled blood samples were centrifuged at 1,000 ×g for 30 min. Plasma was immediately collected in Eppendorf tubes and stored at−80 °C until further analyzed.

### Cell Culture and Treatments

In all cell experiments, bovine aortic endothelial cells (BAECs) were utilized. Proliferating BAECs were purchased from Cell Applications (San Diego, CA). Cells were cultured in Endothelial Growth Medium (Cell Applications, San Diego, CA) and maintained in a humidified atmosphere at 37 °C and 5% CO_2_. Before starting experiments, cells were adapted to grow in low glucose Dulbecco’s Modified Eagle Medium (DMEM) (Gibco Fisher Scientific, Waltham, MA). In addition, the medium was also supplemented with 10% FBS, 1% penicillin/streptomycin, and 1% l-glutamine. When cells reached 80% confluency, they were serum-starved overnight in 0.2% FBS. In hyperglycemia conditions d-glucose (Sigma, St. Louis, MO) was added to the cell medium at a concentration of 25 mmol/L. Cells were treated with L-citrulline (2.5 mmol/L) (Sigma, St. Louis, MO). All experiments were performed with cells from passage 3–9.

### Arginase Activity

Arginase activity was measured using a colorimetric determination method of urea production from L-arginine as described previously (Corraliza et al., 1994). In brief, 25 μL of supernatant was heated with MnCl_2_ (10 mmol/L) for 10 min at 56 °C to activate arginase. The mixture was then incubated with 50 μL L-arginine (0.5 M, pH 9.7) for 1 h at 37 °C to hydrolyze L-arginine.

The hydrolysis reaction was stopped with acid, and the mixture was then heated at 100 °C with 25 μL α-isonitrosopropiophenone (9% α-ISPF in ethanol) for 45 min. The samples were kept in the dark at room temperature for 10 min, and absorbance was measured at 540 nm.

### Nitric Oxide Measurement

#### Plasma Samples

Nitric oxide (NO) was measured in plasma samples as nitrite after enzymatic conversion by nitrate reductase. Nitrite measurement was determined in a microplate assay using Griess reagent utilizing a Griess Reagent System (Promega, Madison, WI) according to the manufacturer instructions. Plasma (100 μL) was mixed with an equal amount of 1% sulfanilamide in 5% phosphoric acid and incubated at room temperature for 5 min. Then 100 μL of 0.1% N-1-naphthylethylenediamine dihydrochloride in water was added, and the mixture was incubated for an additional 5 min. The absorbance at 550 nm was read with a microplate reader. NO_2−_ concentrations were determined using sodium nitrite as a standard.

### Cell Experiments

To measure NO in cell experiments, nitrite (NO_2_) the stable breakdown product of NO in the cell conditioned medium was analyzed using NO specific chemiluminescence [11]. After cells were treated, medium was replaced with fresh Dulbecco’s Modified Eagle’s medium (DMEM) (Gibco Fisher Scientific, Waltham, MA) for 30 min, and medium aliquots were then collected for basal reading. Cells were then exposed to the calcium ionophore ionomycin (Sigma Aldrich, St. Louis, MO) (1 μmol/L) for 30 min and medium samples were collected. In brief, samples containing NO_2_ were injected in glacial acetic acid containing sodium iodide. NO2 is quantitatively reduced to NO under these conditions, which can be quantified by a chemiluminescence detector after reaction with ozone in an NO analyzer (Sievers, Boulder, CO). The amount of NO generated is calculated as the difference in basal and ionomycin-stimulated NO levels.

### Vascular Function

Mouse aortas were harvested from C57BL/6J wild-type mice. After euthanizing the animal, aorta was dissected and immediately placed in ice-cold Krebs-Henseleit buffer, cleaned and cut into 2–3 mm segments. Aorta rings were incubated for 24 h in either, low (5 mmol/L), or high glucose (25 mol/L) DMEM media (Gibco Fisher Scientific, Waltham, MA), and maintained in a humidified atmosphere at 37 °C and 5% CO_2_. L-citrulline (2.5 mmol/L, 24 h) was added to the high glucose media in a subset of experiments. Aortic rings were mounted in an oxygenated tissue chamber (Danish Myo Technology) containing Krebs-Henseleit buffer and allowed to equilibrate for 1 h at a resting tension of 5 mN, during which time the buffer was changed every 15 min. After the 1 h equilibration, vessels were contracted with phenylephrine (PE, 10^−6^ M) to test vessel viability. Vessels were then washed and dose-relaxation curves were performed following fully developed contraction to PE (10^−6^ M). The contractile response was measured in each experiment and compared among experimental groups. Endothelium dependent vasorelaxation was tested by adding increasing doses (10^−10^–10^−4^ M) of acetylcholine (ACh) at 3 min intervals. Vessels were washed and equilibrated for 1 h between successive curves. Vasorelaxation responses are expressed as percent of PE-induced contraction.

### Statistical Analysis

Statistical analysis was performed using GraphPad Prism eight Software. Differences in arginase activity and NO production before and after L-citrulline supplementation in patients was determined using a Paired t-test. Differences between treatment groups in cell culture experiments of arginase activity and NO production and vascular function assay were analyzed by one-way ANOVA followed by Tukey’s post hoc test. The correlations between arginase activity levels and glycated hemoglobin (HbA1c) were determined by Pearson’s correlation test.

## Results

### Demographics Data

Table 1 summaries study participants’ demographic data. Twenty five patients participated in this study. There were 12 females and 13 males. Ages ranged between 25 and 65.

**TABLE 1:** Demographic data and clinical measures (HbA1c, FBS) and concomitant disease of individuals enrolled in the study. N = 25. SD, standard deviation. HbA1c, glycated hemoglobin. FBS, fasting blood sugar.

Variables	N = 25
Age (years)	
Mean ± SD	42.7 ± 10.7
Variation	25–65
Gender	
Males	52%
Females	48%
HbA1c- before treatment (% mean ± SD)	7.88% ± 0.82
HbA1c- after treatment (% mean ± SD)	7.54% ± 1.02
FBS (mg/dl) (mean ± SD)	184.6 ± 49.6
FBS (mg/dl) (mean ± SD)	192.2 ± 39.9
Individuals with concomitant health problems (n, %)	
Hypertension	11, 44%
Ischemic heart disease	5, 20%
Heart failure	2, 8%
History of myocardial infarction	4, 16%
History of cerebrovascular accident	1, 5%
Dyslipidemia	12, 48%
Asthma	2, 8%
Rheumatoid arthritis	5, 2%

All patients were diagnosed as diabetic before the intervention, having a glycated hemoglobin (HbA1c) level ranging between (6.6–9.7). Data also show fasting blood glucose (FBS). Any cardiovascular or inflammatory diseases are reported. Antidiabetic medications received by patients are detailed in Supplementary Table S1.

Although there seems to be a modest decrease in HbA1c after treatment, this change was not significant. Also, fasting blood glucose did not change with L-citrulline supplementation.

### Arginase and HbA1c

**FIGURE 1:**
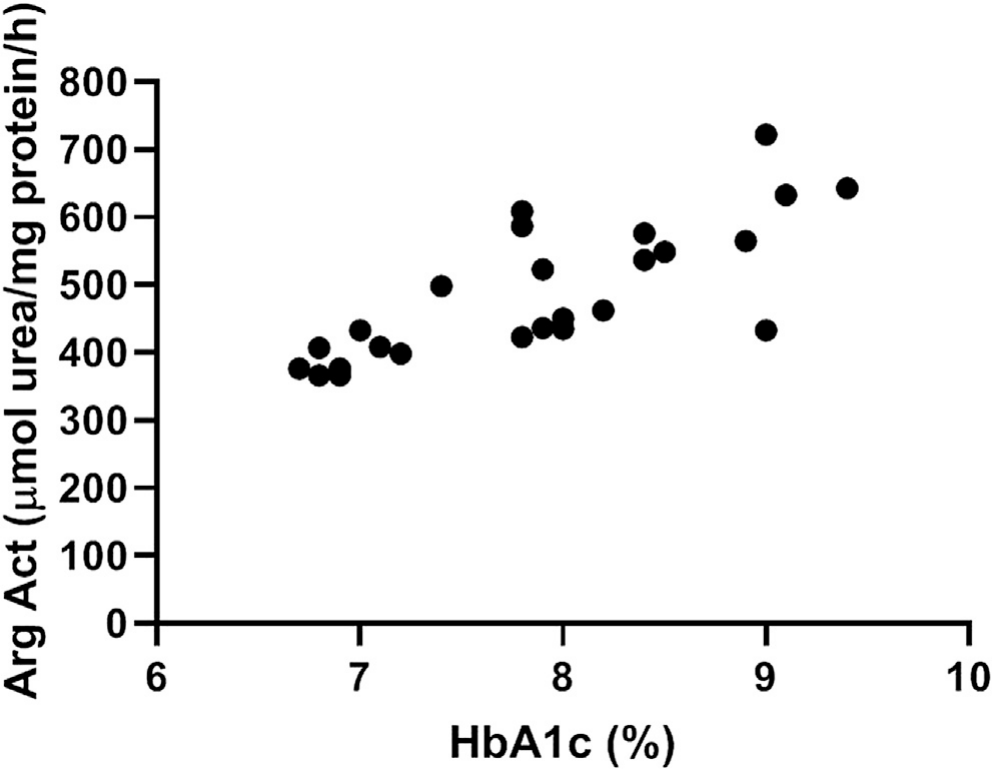
Plasma arginase activity correlates positively with HbA1c levels. Data shows correlation between arginase activity levels measured in plasma of diabetic patients and their glycated hemoglobin Hb1Ac. N = 25. Pearson’s correlation analysis was performed. Pearson R = 0.77, Confidence interval =(0.54–0.89), *R* ^2^ = 0.60.

Our data show a significant correlation between arginase activity levels measured in plasma of diabetic patients and their glycated hemoglobin Hb1Ac (Figure 1). Pearson R = 0.77, Confidence interval (0.54–0.89), *R* ^2^ = 0.60.

### Arginase Activity in Plasma

**FIGURE 2:**
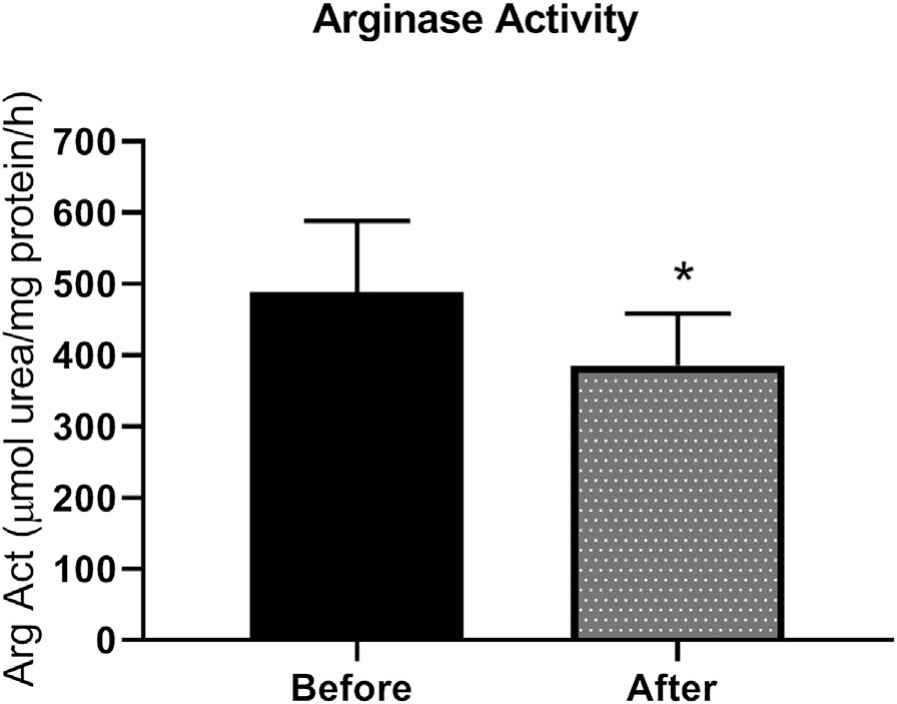
arginase activity is reduced L-citrulline supplementation in T2DM patients. L-citrulline treatment resulted in a decrease in arginase activity in T2DM patients. N = 25. Paired t-test was performed. *p < 0.05.

Patients with type 2 diabetes received L-citrulline (1000 mg twice a day) for a period of one month. Plasma arginase activity was determined before and after stopping treatment. Results show a 21% significant decrease in arginase activity of about 100 µmole urea/mg protein (Figure 2).

### Nitric Oxide in Plasma

**FIGURE 3:**
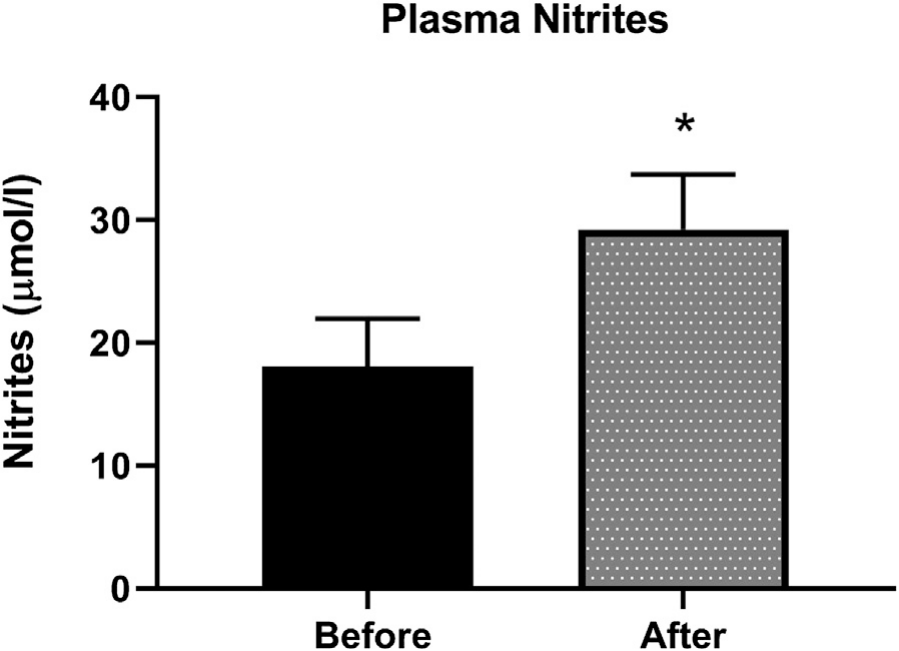
Plasma nitrites level is increased after L-citrulline supplementation in T2DM patients. L-citrulline treatment resulted in an increase in plasma nitrites in T2DM patients. N = 25. Paired t-test was performed, *p < 0.05.

Nitrite, the stable metabolite of NO, was detected by the Griess method in plasma samples. Figure 3 shows an increase of 38% in nitrite levels in plasma, indicative of increased NO production in patients after L-citrulline supplementation.

### Arginase Activity in Bovine Aortic Endothelial Cells

**FIGURE 4:**
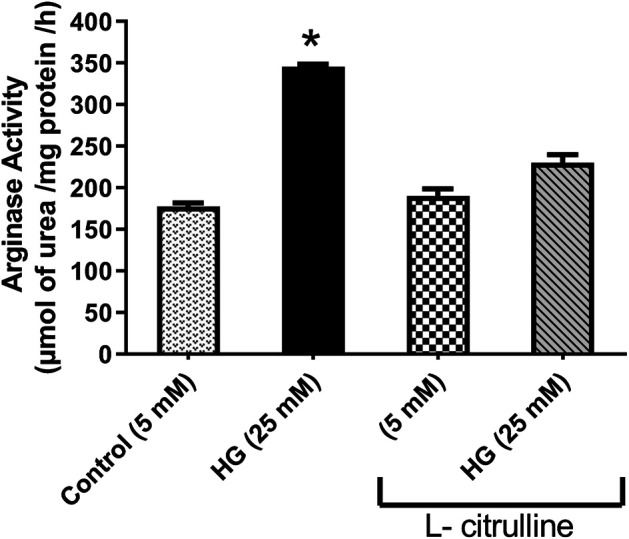
L-citrulline pretreatment prevents high glucose-induced elevation in arginase activity in endothelial cells. Arginase activity in BAECs representing an average of three separate experiments done in triplicate. High glucose treatment caused a 50% increase in arginase activity. Pretreatment with L-citrulline (2.5 mM) inhibited the high glucose-induced increase in arginase activity. Statistical analysis performed using one-way ANOVA followed by Tukey’s post hoc test *p < 0.05.

BAECs were incubated in conditions of high glucose (25 mmol/L) for three days with changing the medium every day. These cells were compared to cells grown in normal glucose concentration (5 mmol/L). Results show that high glucose treatment caused a 50% increase in arginase activity. Pretreatment with L-citrulline (2.5 mM) inhibited the high glucose-induced increase in arginase activity bringing it back to levels similar to that of control (Figure 4). Arginase activity assay was validated using a highly potent arginase inhibitor 2(S)-amino-6-boronohexanoic acid (ABH) as a negative control, though the data is not shown.

### Nitric Oxide Production in Bovine Aortic Endothelial Cells

**FIGURE 5:**
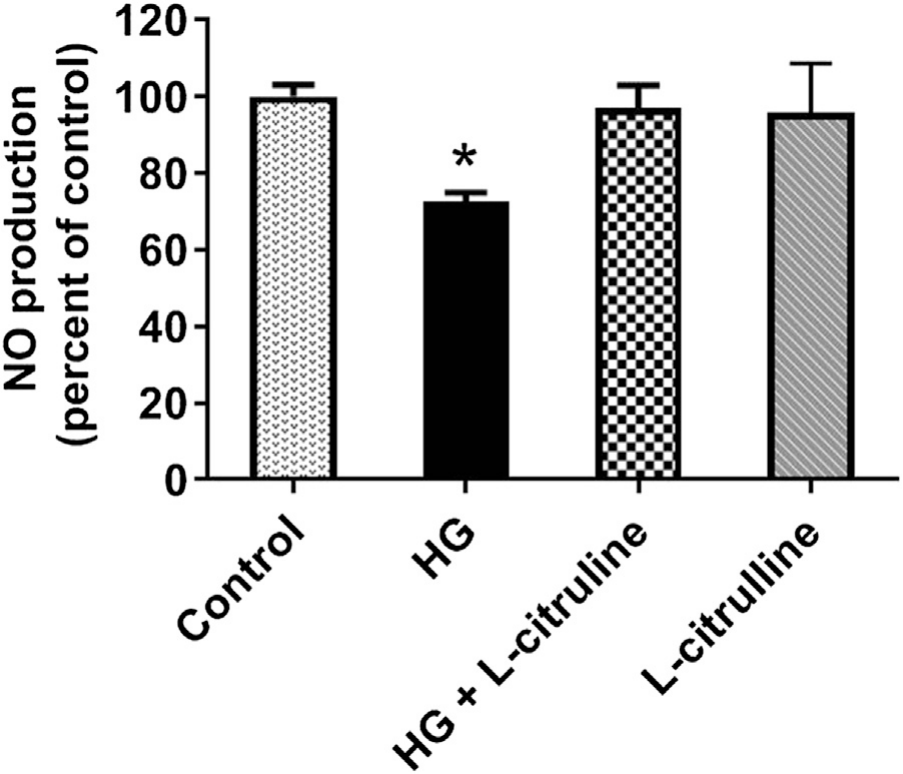
L-citrulline pretreatment prevents high glucose-induced decrease in arginase activity in endothelial cells. NO production percentage in BAECs representing an average of three separate experiments done in six replicates. NO production decreased by 30% as compared to ionomycin stimulated control. Pretreating endothelial cells L-citrulline (2.5 mM), restored NO production to normal levels. Statistical analysis performed using one-way ANOVA followed by Tukey’s post hoc test *p < 0.05.

BAECs were incubated in conditions of high glucose (25 mmol/L) for three days with changing the medium every day. These cells were compared to cells grown in normal glucose concentration (5 mmol/L). Ionomycin-stimulated NO production was calculated in reference to basal reading in high glucose vs. control (normal glucose) treated cells. NO production decreased by 30%. Pretreating endothelial cells with L-citrulline (2.5 mM), restored NO production to normal levels. L-citrulline (2.5 mM) treatment alone in normal glucose conditions (5 mmol/L) had no effect on stimulated NO production (Figure 5).

### *Ex-Vivo* Vascular Function

**FIGURE 6:**
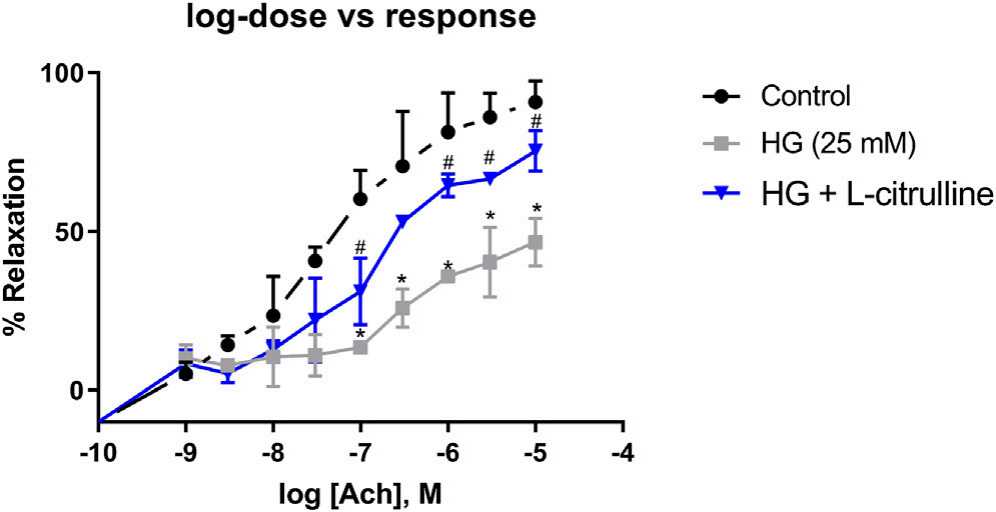
Vascular Endothelial Dysfunction caused by high glucose treatment is mitigated with L-citrulline. Aortic endothelium dependent relaxation to acetylcholine (ACh). Solid black line indicates responses in control rings. Gray tone line indicates responses in high glucose treated rings. Blue lines indicate response in high glucose + L-citrulline treated rings. Mean aortic vasorelaxation responses of n = 4 calculated as a percentage of respective control responses. Statistical analysis performed using one-way ANOVA followed by Tukey’s post hoc test. HG; high glucose, Cont; control. *HG vs control *p* < 0.05. # vs HG + L-citrulline *p* < 0.05.

Mouse aortic rings were incubated in high glucose conditions with and without L-citrulline (2.5 mM) for 24 h. Endothelial dependent vasorelaxation in response to increasing doses of Acetylcholine was impaired under high glucose conditions. Impairment of endothelial function was partially restored in blood vessels cotreated with L-citrulline (Figure 6).

## Discussion

Vascular endothelial dysfunction has been shown to be an early sign of atherosclerosis. It has been also shown to be among the vascular complications in type 2 diabetes mellitus and is an important cause of increasing morbidity and mortality in these patients (Deedwania, 2004). Recently, arginase has been shown to be elevated in an array of cardiovascular conditions such as hypertension, ischemic reperfusion injury and also the vascular complications of diabetes mellitus (Lucas et al., 2014). Nitric oxide (NO) the magic molecule is produced in the vasculature by endothelial nitric oxide synthase (NOS) and is essential for normal vascular function. Endothelial dysfunction leading to decreased blood flow is strongly implicated in the complications of diabetes. Arginase, an enzyme which metabolizes L-arginine to urea and ornithine, can compete directly with NOS for L-arginine. Hence increases in arginase activity can decrease arginine levels, reducing its availability to eNOS, thereby, decreasing NO production and increasing superoxide production. Increases in oxidative stress and decreased production of NO by eNOS in vascular endothelial cells are considered to be responsible for vascular dysfunction (Romero et al., 2012). Enhanced arginase activity also contributes to cell proliferation and collagen formation by elevating polyamine and proline levels respectively, leading to vascular stiffness (Bhatta et al., 2015). Previously, it has been shown that increased arginase expression and activity contribute to vascular endothelial cell dysfunction in diabetes (Romero et al., 2012).

Arginase is elevated in diabetic patients and correlates with vascular disease incidence. In an earlier study published by our group, we showed that plasma arginase activity is 1.7 fold higher in diabetic patients vs. healthy non-diabetic controls (Shatanawi & Momani, 2017). We, and others, have also shown that arginase activity and expression are elevated under high glucose conditions which can lead to limited NO levels and vascular dysfunction (Romero et al., 2012). Additionally, arginase inhibition has been shown to have a beneficial effect on vascular function in diabetic patients (Kovamees et al., 2016). In this study we examined the effect of L-citrulline supplementation on arginase activity in diabetic patients. Our results show that L-citrulline treatment caused a significant decrease in arginase activity. Additionally, we have shown that NO production is enhanced in response to L-citrulline supplementation. We have confirmed these findings in a cell model of high glucose. We have seen that high glucose treatment of endothelial cells resulted in an increase in arginase activity. This increase was suppressed when cells were pretreated with L-citrulline. These effects were associated with a decrease in NO production in response to HG that was completely restored with L-citrulline supplementation.

Work by others have shown an important role of L-citrulline in enhancing NO production in the vasculature. Mori et al. have shown that L-citrulline induces retinal blood vessel dilation through enhancing NO production (Mori et al., 2015). Moreover L-citrulline resulted in restoring nitric oxide level and cellular uptake at the brain capillary endothelial cell line (Lee & Kang, 2018). The benefits of L-citrulline on the cardiovascular system are numerous and have mostly been shown to be implicated with activation of the NO pathway (Romero et al., 2006).

While it seems that L-arginine supplementation would be more beneficial for the vascular system, since it is the direct substrate for NOS, several studies, have shown that the benefits of L-citrulline outweigh those of L-arginine in enhancing NO in the vasculature (Romero et al., 2006). Our study has added an important evidence in a patient sample on the effect of this amino acid on vascular NO production. Also, we elucidated another mechanism of its effect that is by inhibiting arginase.

L-citrulline is a natural amino acid that is simply available in watermelon juice and rind (Rimando & Perkins-Veazie, 2005). The benefits of L-citrulline in diabetes are through supporting the cardiovascular system by enhancing NO and by inhibiting excessive arginase activation, further augmenting production of NO through providing more L-arginine for the NOS pathway. Others have reported that L-citrulline possesses an inhibitory effect on arginase and have beneficial effects on enhancing organ perfusion and arginine availability under conditions with enhanced arginase activity (Romero et al., 2006).

Furthermore, we have shown that L-citrulline treatment to blood vessels led to an improvement in vascular endothelial function. Romero et al. studied vascular endothelial function in aorta from arginase knockout diabetic mice. Their work showed enhanced endothelial dependent vasorelaxation, indicating the benefits of reduced arginase expression on restoring vascular function in diabetes (Romero et al., 2012). Other studies have shown similar effects through the use of arginase inhibitors in maintaining normal vascular function in models of hypertension and diabetes (Bagnost et al., 2010). Interestingly, the use of an arginase inhibitor, Nω-hydroxy-nor-L-arginine (NOHA) improved microvascular endothelial function in patients with type 2 diabetes mellitus (Kovamees et al., 2016).

The use of arginase inhibitors has recently shown promising evidence in treating conditions of vascular dysfunction in an array of cardiovascular conditions. There are several inhibitors of arginase used for investigational purposes. None of which is in the market yet. N-Hydroxy-nor-L-arginine (nor-NOHA) was in a clinical trial for treatment of ischemia-reperfusion Injury. Arginase inhibition protected against endothelial dysfunction induced by ischemia-reperfusion injury. Other inhibitors of high potency include, S-(2-boronoethyl)-l-cysteine BEC and 2(S)-amino-6-hexanoic acid (ABH). Although the aforementioned inhibitors are highly potent, they have not been studied clinically (Steppan et al., 2013; Kovamees et al., 2016).

The use of a natural arginase inhibitor as L-citrulline provides the benefits of arginase inhibition while keeping to a minimum side effects that could arise from the use of chemical compounds. Adding to that, the pleiotropic effects of L-citrulline on enhancing vascular and metabolic health through other pathways (Kaore et al., 2013).

Our work represents the first clinical study on the effects of L-citrulline on the arginase pathway and its impact on NO production in diabetic patients. We believe this work would be a cornerstone study that future research will build on in the important field of vascular health.

## Data Availability Statement

The raw data supporting the conclusions of this article will be made available by the authors, without undue reservation, to any qualified researcher.

## Ethics Statement

The studies involving human participants were reviewed and approved by The University of Jordan Hospital Institutional Review Board. The patients/participants provided their written informed consent to participate in this study. The animal study was reviewed and approved by The Universiy of Jordan deanship of scientific research.

## Author Contributions

All authors contributed significantly to this project. AS participated in design, analysis and writing the manuscript. MSM participated in collecting patient data and samples. RA participated in collecting samples, analysis of data and writing manuscript. MHH participated in sample collection and analysis. MNG participated in design of experiments and writing of the manuscript.

## Funding

This work was supported by The University of Jordan Deanship of Scientific Research (to AS, MM, and MG). The clinical data was part of a clinical trial with Identifier NCT03358264.

## Conflict of Interest

The authors declare that the research was conducted in the absence of any commercial or financial relationship that could be construed as a potential conflict of interest.
