# Supplementary material for: L-Citrulline Supplementation Increases Plasma Nitric Oxide Levels and Reduces Arginase Activity in Patients With Type 2 Diabetes
Source: Front Pharmacol. 2020 Dec 22;11:584669. doi: 10.3389/fphar.2020.584669 (PMC7783447; doi:10.3389/fphar.2020.584669)
Supplement: Supplementary file 1 [file datasheet1.docx]

| Diabetes medications received by patients | N=25 |
| --- | --- |
| Metformin | 23, 92% |
| Glimepiride | 12, 48% |
| Sitagliptin | 7, 28% |
| Rosiglitazone | 5, 20% |
| Atorvastatin | 12, 48% |

**Table 1 Suppl.** Antidiabetic drugs received by patients in the study with percentage of patients for each medication.
